# Is the New Langya virus in China a threat to global health? A short communication

**DOI:** 10.1097/MS9.0000000000000322

**Published:** 2023-03-14

**Authors:** Olivier Uwishema, Sanobar Shariff, Anushree Rai, Sara Arab, Aderinto Nicholas, Lama Uweis, Mortada Abbass, Rayyan El Saleh, Irem Adanur, Jack Wellington

**Affiliations:** a Oli Health Magazine Organization, Research and Education, Kigali, Rwanda; b Clinton Global Initiative University, New York, New York, USA; c Faculty of Medicine, Karadeniz Technical University, Trabzon, Turkey; d Yerevan State Medical University, Yerevan, Armenia; e Chhattisgarh Institute of Medical Sciences, Bilaspur, Chhattisgarh, India; f Faculty of Medicine, Beirut Arab University, Beirut, Lebanon; g Department of Medicine and Surgery, Ladoke Akintola University of Technology, Ogbomoso, Nigeria; h Faculty of Medicine, Cardiff University School of Medicine, Cardiff University, Cardiff, UK

**Keywords:** China, henipavirus, Langya virus, LayV, New Langya virus

## Abstract

The recently detected virus in eastern China in 2018 led to some health concerns, especially with the global trend of spreading viruses. As a new RNA-detected genus of the henipavirus family was found in Eastern China, the number of patients affected has reached 35 through zoonotic spread, with symptoms ranging from simple fever to fatal affection of vital organs such as the brain, liver, and kidneys. Researchers have found that shrew animals might be a potential reservoir for the Langya virus; however, data is still limited regarding human-to-human transmission. Current efforts by the Chinese Health Ministry and the Taiwan Centers for Disease Control and Prevention to deduct the spread of the virus and track its origin by trying to sequence the disease genome are evident. With all this in mind, the recommendation to face this new novel virus revolves around protecting the most vulnerable population at risk of being infected, such as farmers, and preventing the spread of the virus. Efforts must be directed toward screening animals for henipavirus and diving more deeply into the etiology of how this virus has spread to humans to help understand the spread of zoonotic viruses in the future.

## Introduction

Infectious disease specialists have long warned that environmental damage and climate change will increase the risk of ‘zoonotic spillovers’, or the spread of infections from animals to humans. In the midst of current efforts worldwide to tackle the ongoing monkeypox virus outbreak and coronavirus disease 2019 (COVID-19) pandemic, a new animal virus known as the Langya henipavirus (LayV) has been discovered in humans in Eastern China^1^. The virus is closely associated with two other henipaviruses that are documented to infect people, Nipah virus (NiV)^2^ and Hendra virus (HeV), which cause severe respiratory diseases and can be fatal, despite the fact that experts have mentioned that the risk for dissemination among humans is low^1^. Langya virus (LayV) is a newly identified virus among the *Henipavirus* genus. According to the Centers for Disease Control and Prevention, five other viruses were previously identified in this genus^3^. HeV and NiV are classified as humanly pathogenic and highly fatal, whereas the Cedar, Ghanaian Bat, and Mojiang viruses are not known to cause human illnesses^4^. LayV was first identified in China in December 2018, when a 53-year-old woman presented to the hospital complaining of fever^4^. After taking a swab sample, the patient was said to be infected with a novel virus from the henipavirus family^5^. According to the *New England Journal of Medicine*, this newly identified pathogen is mostly related to the Mojiang virus^5^. Since its first emergence, the LayV has infected around 35 patients from the Henan and Shandong provinces of China; 26 of them have isolated the LayV infection^5^. All 26 patients complained of fever, and about half of them complained of fatigue, anorexia, and cough^5^. The other less frequently encountered symptoms and abnormalities were headache, nausea and vomiting, thrombocytopenia and/or leukopenia, myalgia, and abnormal liver and/or kidney function^5^.

It is worth mentioning that scientists have limited information regarding the mode of transmission of LayV^6^. However, some researchers postulate that the virus is carried by shrews, which may infect humans either directly or through an intermediate host animal^7^. This assumption was made after testing 25 different wild animals. Results showed that 27% out of the 262 tested shrews were carrying LayV, which is the highest share among other species^3^,^4^. Moreover, nine patients reported direct contact with close family members with no evidence of disease transmission^3^. Thus, researchers suggest that it is less likely for the virus to be transmitted from one person to another. On the other hand, this sample number is too small to rule out person-to-person transmission^3^.

### Epidemiology and outbreak of Langya virus in China

**Figure 1:**
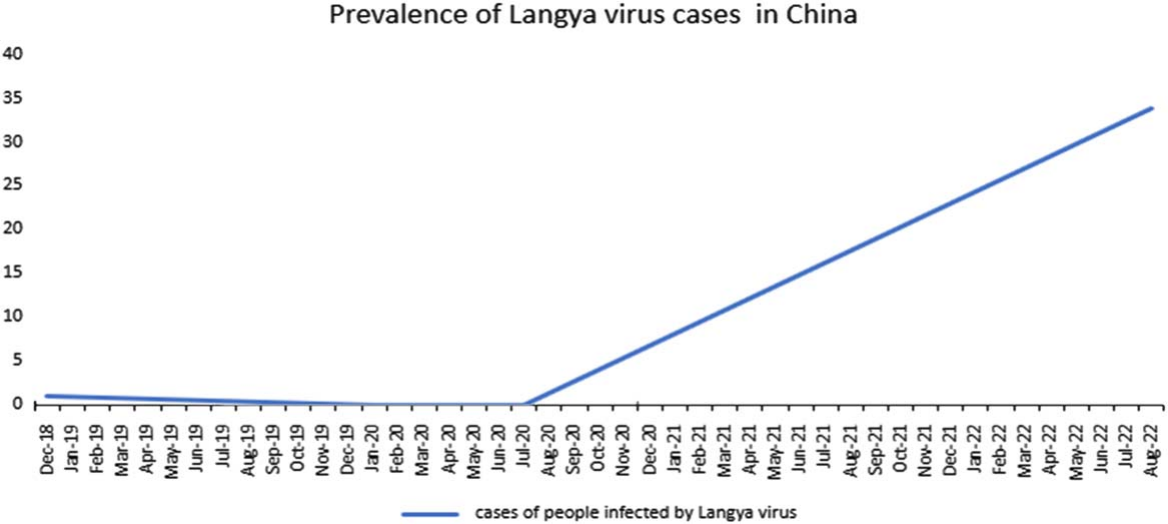
Prevalence of Langya virus cases in China.

In December 2018, as part of a program to monitor humans who experienced fevers after encountering animals, researchers used a sample from a throat swab to discover the novel virus ‘via metagenomic analysis and subsequent virus isolation’.^8^,^9^ Following the discovery of the LayV, scientists observed samples from people who developed fevers after being exposed to animals over the following 2 years^8^,^9^. They discovered the LayV in an additional 34 people during this time^9^. These 35 individuals, who have been infected with the ‘Langya’ henipavirus (LayV), were distributed in the Chinese provinces of Henan and Shandong (Fig. 1). Of these, only 26 had the LayV infection (no other viruses)^10^. According to a study conducted by the Beijing Institute of Microbiology and Epidemiology, no cases of the LayV infection occurred between January 2020 and July 2020, in the first year of the pandemic^11^. However, the study indicated that from July 2020 onward, there were more than 11 occurrences of Langya^10^. Between 2018 and 2021, following normal patient screening for possible zoonotic infections in three hospitals in eastern China, researchers discovered LayV^11^. This new virus appears to have distant relatives that are relevant in humans: the NiV and the HeV^10^. In Queensland, the HeV was first identified in 1994. Globally, the NiV is more prominent, and Bangladesh is typically the site of outbreaks^8^.

### Etiology of Langya virus

To determine which animal species was the source of the LayV, the researchers tested several animals for the presence of the virus^12^ and suggested that the shrew may be a natural reservoir of the LayV^13^. The family of RNA viruses has been classified into a genus referred to as henipavirus. It is related to HeV and NiV^13^. Both NiV and HeV occur naturally in bats (*Pteropus* spp.). Their transfer to mammals eventually led to their ability to infect human beings^14^. The severity of infection can range from very mild to fatal encephalitis^14^. The primary outbreak in Malaysia and Singapore was reported in people who had close contact with pigs^14^. However, the more recent outbreaks have been due to food contaminated with the urine or saliva of infected bats^14^. Little is known about this new virus, and therefore the currently reported cases are likely to be the tip of the iceberg^14^. At this stage, there is no indication that the virus can transmit from person to person^14^. More research is required to determine the origin of the zoonotic virus, how severe the infection could be, and how it spreads^14^.

### Current efforts to mitigate the Langya virus in China

The current Langya outbreak comes against a background of debates around the origin of the severe acute respiratory syndrome coronavirus 2 virus. One of the several points raised in the scientific and public communities is the slow rate at which the Chinese government alerted the world about the emergence of an unusual infection^15^,^16^. On 4 August 2022, barely days after the outbreak, a leading English journal, the *New England Journal of Medicine*, published a paper detailing the new virus’s details^16^. Though little is known about the virus, the paper served as an early alert within the scientific community^16^. However, this is not the first time the LayV has infected people^16^. In 2018, about 35 cases were detected, but it was not until August 2022 that they were identified^16^. The first cases were detected in the background of a strict routine surveillance system at three Chinese hospitals between 2018 and 2021^16^. No mortality has been recorded from the infection^16^. This preparation further enhances the swift response of China, even before the virus became a concern^16^. The Chinese public health system has activated a tracking system to identify and curb the spread of viral infection^16^. Neighboring Island, Taiwan, is also monitoring the viral infection concurrently. The Taiwan Centers for Disease Control has started the genomic sequencing of the virus^16^. These are reasonable steps toward mitigating and containing the infection within China. The country must implement a robust system to sustain and learn from the COVID-19 pandemic^16^. There is a paucity of scientific evidence regarding the mode of transmission and other characteristics of the viral infection. Nevertheless, it is expedient that the Chinese health system activate health protocols consistent with past viral respiratory infections of zoonotic origin^16^.

## Recommendations

**Figure 2:**
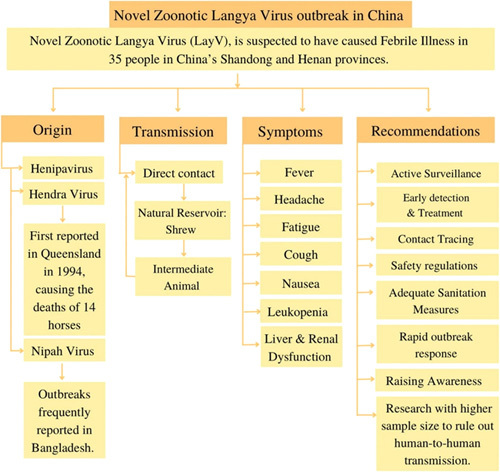
A summary of the New Langya Virus identified in China and recommendation to curb the disease.

Since farmers made up the majority of the 35 cases recorded, comprehensive sanitation measures should be adopted in the field of animal husbandry. Disease surveillance revealed no shared exposure sources or close contact between infected individuals, indicating that human infection may have happened sporadically, according to the research teams^17^. There is currently no evidence that the virus may transfer from human to human^18^. The discovery of a novel virus in dozens of individuals in eastern China may not be the start of the next pandemic, but it does highlight how readily viruses may spread undetected from animals to people, according to scientists^19^. There is, however, no proof that the LayV is contagious or that it was the root of a local outbreak of related cases, according to the studies^19^. To exclude human-to-human dissemination, more research on a broader proportion of patients is required^19^. The two provinces where the virus was discovered, as well as other parts of China, should perform additional research screening for Langya henipavirus (Fig. 2)^18^. Critical questions need to be addressed regarding how prevalent the new virus may be in nature, how it is affecting people, and how threatening it is to public health. The necessity for methodical investigations to explain the pathogenesis of the virus, along with the broader issue of anthroponomic infections with viruses from animals, was suggested by recent findings, which also indicated a huge number of undiagnosed diseases spreading from animals to people^19^.

## Conclusion

Since the novel LayV was detected in late 2018, dozens of patients have been infected with little information on the origin of reported cases and the viral exposure through interhuman transmission. This has left the Chinese health care system with concerns concerning tracking the virus and sequencing its genome. Unfortunately, this new virus lacks a targeted treatment and a protective vaccine, despite the fact that doctors are trying different antivirals to treat the symptoms. Prevention seems to be the key to avoiding this viral conflict. Moreover, experts have mentioned that the likelihood of this disease transforming into a pandemic is still little to none. However, with the observed COVID-19 pandemic, more efforts must be initiated to target the origin and curb the spread of the infection. We highly recommend implementing a targeted, robust system to face this virus and putting some effort into activating efficient health policies to deduct the viral load from known zoonotic reservoirs. Appropriate investment in vaccine-development research might save countries from potential outbreaks in vulnerable sites such as farms and sites where human–animal interaction occurs.

## Ethics approval

Not applicable.

## Consent for publication

Not applicable

## Sources of funding

We have not received any financial support for this manuscript.

## Authors’ contribution

O.U.: conceptualization, project administration, writing – review and designing, reviewed and edited the first draft, and reviewed and edited the final draft. J.W.: reviewed and edited the second draft. S.S.: reviewed and edited the third draft. All authors contributed in data collection and assembly, manuscript writing, final approval of manuscript. Figure 1 was drawn and analyzed by authors S.A. and O.U. Figure 2 was drawn and analyzed by authors A.R. and O.U.

## Conflicts of interest disclosure

The authors declare no conflicts of interest.

## Research registration unique identifying number (UIN)

1. Name of the registry: not applicable.
2. Unique identifying number or registration ID: not applicable.
3. Hyperlink to your specific registration (must be publicly accessible and will be checked): not applicable.

## Guarantor

Olivier Uwishema: principal investigator.

## Data availability statement

Not applicable.

## Provenance and peer review

Not commissioned, externally peer reviewed.
